# How Printing Affects the Health

**Published:** 1882-02

**Authors:** 


### HOW PRINTING AFFECTS THE HEALTH.

Years ago there was a notion prevalent among those who were but partially informed upon the question, that the printing business was essentially detrimental to health. There was a tradition about the absorption of poison from the constituents of which type metal was composed. This was and is true in so far as it asserts the poisonous nature of some of the constituent parts of type metal; but that these poisons should necessarily be absorbed into the system of one who handles type is simply absurd. Printers who have such habits of cleanliness and sobriety, as a decent respect for one's self and the opinion of others might be expected to dictate, may follow their calling for years without experiencing any further damaging effects upon their health than what will result from close application to any sort of hard work. More "poison" is absorbed by the printer when taking observations of his little finger through the bottom of a glass than in any other way.—*The Chicago Specimen.*

